# Inter-epidemic Transmission of Rift Valley Fever in Livestock in the Kilombero River Valley, Tanzania: A Cross-Sectional Survey

**DOI:** 10.1371/journal.pntd.0002356

**Published:** 2013-08-08

**Authors:** Robert D. Sumaye, Eveline Geubbels, Edgar Mbeyela, Dirk Berkvens

**Affiliations:** 1 Ifakara Health Institute, Ifakara, Tanzania; 2 Department of Infectious and Parasitic Diseases, Faculty of Veterinary Medicine, University of Liege, Liege, Belgium; 3 Department of Biomedical Sciences, Institute of Tropical Medicine, Antwerp, Belgium; Centers for Disease Control and Prevention, United States of America

## Abstract

**Background:**

In recent years, evidence of Rift Valley fever (RVF) transmission during inter-epidemic periods in parts of Africa has increasingly been reported. The inter-epidemic transmissions generally pass undetected where there is no surveillance in the livestock or human populations. We studied the presence of and the determinants for inter-epidemic RVF transmission in an area experiencing annual flooding in southern Tanzania.

**Methodology:**

A cross-sectional sero-survey was conducted in randomly selected cattle, sheep and goats in the Kilombero river valley from May to August 2011, approximately four years after the 2006/07 RVF outbreak in Tanzania. The exposure status to RVF virus (RVFV) was determined using two commercial ELISA kits, detecting IgM and IgG antibodies in serum. Information about determinants was obtained through structured interviews with herd owners.

**Findings:**

An overall seroprevalence of 11.3% (n = 1680) was recorded; 5.5% in animals born after the 2006/07 RVF outbreak and 22.7% in animals present during the outbreak. There was a linear increase in prevalence in the post-epidemic annual cohorts. Nine inhibition-ELISA positive samples were also positive for RVFV IgM antibodies indicating a recent infection. The spatial distribution of seroprevalence exhibited a few hotspots. The sex difference in seroprevalence in animals born after the previous epidemic was not significant (6.1% vs. 4.6% for females and males respectively, p = 0.158) whereas it was significant in animals present during the outbreak (26.0% vs. 7.8% for females and males respectively, p<0.001). Animals living >15 km from the flood plain were more likely to have antibodies than those living <5 km (OR 1.92; 95% CI 1.04–3.56). Species, breed, herd composition, grazing practices and altitude were not associated with seropositivity.

**Conclusion:**

These findings indicate post-epidemic transmission of RVFV in the study area. The linear increase in seroprevalence in the post-epidemic annual cohorts implies a constant exposure and presence of active foci transmission preceding the survey.

## Author Summary

Rift Valley fever (RVF) is an arthropod-borne viral disease that affects people, livestock and wild animals. It occurs mostly in Africa, and epidemics have been reported in the Arabian Peninsula. RVF is transmitted to humans and animals by mosquitoes, but people can also get the infection through direct contact with blood or tissues of infected animals. The disease occurs in epidemic form in a cycle of 5–15 years, but some reports also indicate occurrences of the disease during non-epidemic periods. We report here inter-epidemic period transmission of RVF in livestock population, evidenced by demonstration of RVFV antibodies in animals that were born after the 2006/07 RVF outbreak in Tanzania and demonstration of immunoglobulin M (IgM), a short lived class of antibodies, following infection by RVF virus in 9 samples. We have also identified hotspots of transmission in the study area, with exposure being higher away from the main flood plain. There was a linear increase in percent seropositivity from 1 year olds to age 5 years, implying a possible annual challenge.

## Introduction

Rift Valley fever (RVF) is known to occur in outbreaks in cycles of 5–15 years in the Eastern Africa region and the Horn of Africa, following unusual high precipitations that lead to sustained flooding [1], [2]. In recent years, evidence of RVF transmission during the inter-epidemic periods in some parts of the African continent has increasingly been reported [3]–[5]. The inter-epidemic transmissions generally pass undetected clinically, but can be revealed where active serological surveillance is regularly done in either livestock or human populations [4], [6], [7].

Rift Valley fever is a mosquito borne viral zoonosis that affects both livestock and wild ruminants [4], [5], [8]. It is caused by Rift Valley fever virus (RVFV) belonging to the genus *Phlebovirus* of the family *Bunyaviridae* and in susceptible animals is manifested clinically by high fever, and causes abortion in susceptible pregnant animals irrespective of the gestation period and high mortality in newborn animals [9]. In humans, RVF can be asymptomatic, but can also cause mild illness (associated with headache, fever, muscle and joint pains) or severe illness (associated with hemorrhagic fever, encephalitis or ocular disease) [10]–[12].The disease was first described in the early 1910s and the aetiological agent was isolated in the 1930s in Kenya [13]. The disease pattern in the Eastern Africa region and the horn of Africa is driven by climatic conditions linked to the El Niño/Southern Oscillation (ENSO) phenomenon, which leads to unusual high rainfall and floods alternated by long dry spells [2]. In other parts of Africa, RVF emerged in relation to the construction of hydroelectric power dams along Senegal river and thereafter established itself as endemic disease [14], [15]. In the Arabian Peninsula RVF was introduced through trading of live animals with countries in the horn of African [16], [17].

RVF maintenance in nature between epidemics both in the mammalian host and vector populations has not been fully explained [18], [19]. This is partly due to the factors driving its maintenance at fine geographical scales that are yet to be described in detail. Such factors interact in diverse ways in different geographical regions of Africa or beyond and may play a crucial role in vector population dynamics and the disease transmission [20].

The disease in humans is primarily an occupational hazard affecting people in close contact with infected animals through blood and body fluids, e.g. livestock keepers, abattoir workers, laboratory workers and veterinarians in the course of taking care and treating sick animals, butchering, or disposing of dead animals and aborted fetuses [21]–[25]. Consumption of animal products such as unpasteurized milk and raw meat has been identified as exposure risk factors in some observational studies [26] but attempt to show infection through oral route in experimental studies had mixed results [27], [28]. People can also acquire the disease through infectious mosquito bites [24], [29].

In the past two decades, RVF outbreaks have been reported twice in Tanzania, with the most recent outbreak occurring in 2006/07. During this outbreak, the disease occurred in 10 of the 21 regions of Tanzania mainland, including Morogoro region where the Kilombero river valley is located, with clinical cases in people and livestock [30]–[32].Given the ecology and climatic factors that support mosquito vectors in the valley we hypothesized a continued transmission of RVFV during the inter-epidemic periods. The goal of our study was therefore to investigate potential inter-epidemic period transmission of RVF in an area experiencing seasonal floods annually. Specifically, the study aimed 1) to establish baseline information on the exposure status to Rift valley fever in livestock as determined by presence of antibodies against RVFV in livestock blood samples during the inter-epidemic period and 2) to assess the relationship between RVF seropositivity, animal characteristics and environmental factors.

## Methods

### Study area

**Figure 1:**
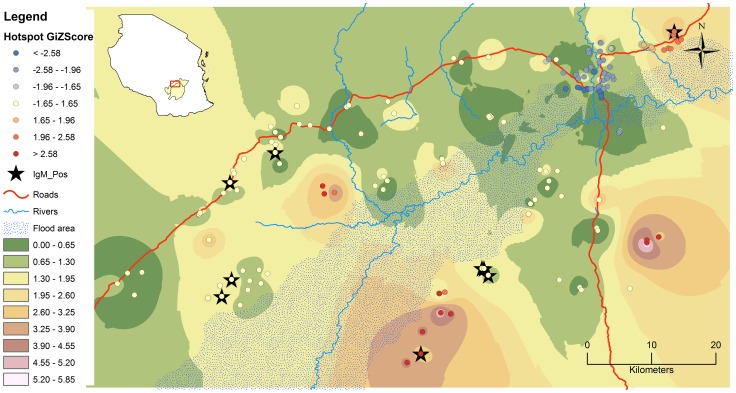
Study area depicting spatial distribution pattern of RVF seroconversion in livestock. The graduated colors indicate output of IDW based on actual number of seropositive animals to RVFV antibodies in all species. The round dots represent location of households from which livestock were sampled and clustering pattern given by Z-scores (red, blue and white colorations indicating hotspot, cold spot and random pattern respectively). The black stars indicate households where IgM positive animals were sampled. The insert is Map of Tanzania showing districts of Kilombero and Ulanga, and location of study site within.

This study was conducted in the Kilombero river valley, which spans Kilombero and Ulanga districts in Morogoro region. The Kilombero valley is a seasonally inundated flood plain up to 52 km wide, at high water, between the densely forested escarpment of the Udzungwa mountains, which rise to 2576 m above sea level (asl) on the north-western side and the grass covered Mahenge mountains, which rise to 1516 m asl on the south-eastern side (Figure 1). The valley receives an average annual rainfall of 1200 mm–1800 mm and temperatures range between 25°C and 32°C. The valley has a diverse ecology and demography with villages consisting largely of numerous distinct groups of houses located on the margins of the flood plain where rice cultivation is the predominant economic activity. Other land use types include hunting, fishing, forestry, pastoral livestock rearing and other crops cultivation. There has been intense malaria transmission in the valley with the main vectors being *Anopheles gambiae* complex and *Anopheles funestus* [33]. Other mosquito species inhabiting the valley, some of which are vectors of RVF virus, include *Culex* spp, *Aedes* spp *and Mansonia* spp [34].

### Design, sampling and data collection

The sero-survey was done from May to August 2011, approximately four years after the end of the 2006/07 RVF outbreak in Tanzania. Blood samples were collected from three livestock populations, namely cattle, goats and sheep from 44 villages in both districts. These villages were selected based on their inclusion in a demographic surveillance system [35], [36] and the proximity to the Kilombero river flood plains (figure 1). In each village four households that kept at least one of the three species, were randomly selected from the list of livestock keepers in that particular village. For each participating household a maximum of 18 samples were collected (i.e. maximum 10 cattle, maximum 4 goats and maximum 4 sheep, the actual numbers sampled depending on the number of a particular species present). The sampling strategy was based on selecting animals of all age groups in order to be able to characterize epidemic and inter-epidemic transmission. The blood samples were collected in 6 ml vacutainer tubes with clot activator, labeled and stored in a cooler box with ice packs while in the field. After coagulation, serum was eluted from the whole blood into a 1.8 ml cryovial tube, labeled and stored in a car fridge until transfer to the laboratory for laboratory analysis. Characteristics of individual animals together with the herd history were obtained through a structured interview with the herd owner.

### Age determination

Individual animal age was estimated using history taking, review of available records on date of birth and dentition. Records were available only from households keeping exotic dairy cattle. Dentition was used in determining age of cattle between 24 to 54 months only [37]. When the above two methods yielded no useful results, age was estimated by probing the head of the household, herd boys and other members of the household for the animal's month/season and year of birth and for female animals also by taking into account number of births and average birth rate for the particular species in the valley.

### Serological assays

To determine the individual animals' longstanding exposure status to previous Rift Valley fever virus (RVFV), a commercial, inhibition enzyme-linked immunosorbent assay (c-ELISA) for the detection of antibodies against RVFV in humans, domestic and wildlife ruminants was used (Biological Diagnostic Supplies Limited, Dreghorn, United Kingdom) [38]. Recent infection was determined using a commercial IgM ELISA kit (Biological Diagnostic Supplies Limited, Dreghorn, United Kingdom) [39].The IgM ELISA test was employed for c-ELISA positive samples only since the c-ELISA detects both IgG and IgM antibodies against RVFV [38].

### Data analyses

The data was analysed in STATA version 12 [Stata Corp., College Station, Texas, USA]. To examine the determinants for RVF seropositivity, first a univariable analysis of individual factors was performed by fitting a logistic regression model with wards and villages as random effects to account for clustering. Variables with p-value <0.25 were selected as potential covariables in the multivariable analysis, where a p-value ≤0.05 was considered statistically significant. Forward model-building was done with subsequent models evaluated against sparser models by means of the Akaike information criterion (AIC). Two-way interactions between variables included in the model were also tested. Lastly, all factors that were dropped in the process of model building were later tested for any confounding effect. Factors were considered a confounder if they led to a change of ≥25% in the coefficient estimates of other determinants.

The spatial analysis of the seropositivity was performed using ArcGIS software version 10 (ESRI, Redlands, USA) using hot spot analysis and inverse distance weighted (IDW) tools. The Getis-Ord Gi* statistic for each feature (household) was computed with the resultant Z-score values indicating where households with either high (hot spot), median (random) or low (cold spot) values cluster spatially. The IDW is a deterministic interpolation model that assigns values to locations where no measurements have been taken to produce a surface pattern, based on how far those locations are to the sentinel locations where measurements have been taken.

### Ethics statement

The blood collection procedure from livestock was performed by a qualified veterinarian following proper physical restraint of animals that ensured both personnel and animal safety. Livestock owners were explained the study purpose and procedures and upon agreeing to participate they provided a written consent prior to study procedures and blood collection from their animals. Ethical approval for this study protocol was obtained from the Institutional Review Board of the Ifakara Health Institute and Medical Research Coordination Committee of the Tanzania's National Institute for Medical Research (permit number NIMR/HQ/R.8a/Vol. IX/1101).

## Results

**Figure 2:**
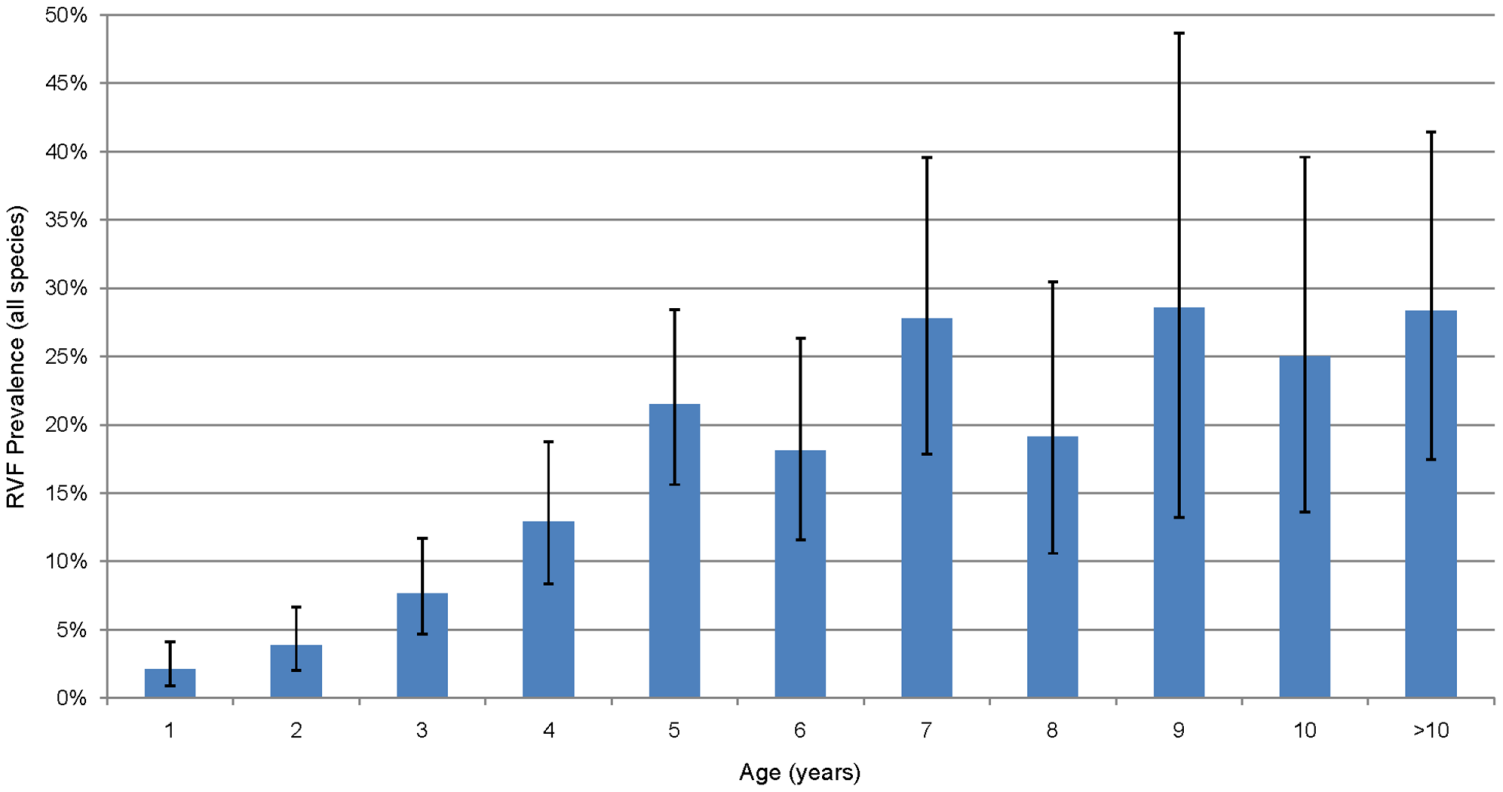
Seropositivity to Rift Valley fever virus in cattle, sheep and goats pooled by age. The error bars indicate 95% confidence intervals of the percentage.

A total of 1680 livestock serum samples were tested by RVF c-ELISA, of which 1234 samples were from Kilombero district and 446 samples were from Ulanga district. Out of the samples tested 970, 455 and 255 were from cattle, goats and sheep respectively. Several potential animal-level risk factors were investigated; table 1 shows the univariable logistic regression model output of the risk factors. The proportion of seropositive animals by c-ELISA was 11.3%. A seroprevalence of 5.5% was recorded among animals that were born after the 2006/07 RVF outbreak (less than 4 years of age), compared to 22.7% in those that were born before and thus present during the outbreak. There was a linear increase in proportion sero-conversion up to age 5, Figure 2. The samples included less than one year olds in which the youngest positive individual was 7 months old at which age the maternally acquired immunity has waned out. There was no significant difference in seropositivity between the different species. The sex difference in seroprevalence in animals born after the previous epidemic was not significant (6.1% vs. 4.6% for females and males respectively, p = 0.158) whereas there was a significant sex difference in seroprevalence in animals that were present during the outbreak (26.0% *vs.* 7.8% for females and males respectively, p<0.001) and overall (table S1).

**Table 1:** Potential animal level risk factors associated with RVF sero-positivity for cattle, goats and sheep in the Kilombero Valley, Tanzania based on a univariable random effect logistic regression model.

Variable	Level	%Positive (n)	OR	95% CI
**Species**	Cattle	11.03 (970)	1	
	Goat	11.86 (455)	1.06	0.74–1.52
	Sheep	11.37 (255)	1.07	0.68–1.69
**Sex**	Female	14.16 (1144)	1	
	Male	5.22 (536)	0.30	0.19–0.46
**Breed**	Exotic	9.54 (220)	1	
	Local	11.61 (1420)	1.23	0.70–2.15
	Mixed	10.00 (40)	0.95	0.28–3.14
**District**	Kilombero	10.69 (1234)	1	
	Ulanga	13.00 (446)	1.17	0.68–2.03
**Present during RVF outbreak**	No	5.55 (1116)	1	
	Yes	22.69 (564)	5.29	3.79–7.38
**Herd composition**	One species	10.27 (360)	1	
	Two species	10.30 (359)	1.08	0.64–1.83
	Three species	12.07 (961)	1.24	0.80–1.92
**Distance to the edge of floods (km)**	<5 km	10.74 (1117)	1	
	5–10 km	14.01 (157)	1.27	0.72–2.21
	10–15 km	7.94 (214)	0.75	0.41–1.37
	>15 km	16.14 (192)	1.62	0.91–2.86
**Feeding practices**	Mixed	4.76 (21)	1	
	Grazing	11.30 (1592)	2.29	0.29–18.18
	Cut & carry	13.43 (67)	2.80	0.31–25.06
**Altitude (m)**	Continuous	n/a	1.007	0.99–1.01

OR = Odds ratio; CI = Confidence interval.

Nine out of c-ELISA positive samples were positive for RVFV IgM antibodies indicating a recent infection. The IgM positive samples originated from 7 villages out of the 44 sampled. The spatial distribution of sero-conversion in the study area exhibited no particular pattern but rather a few hotspots (Figure 1). Each district had an almost equal share of the disease, and this was true for the wards. Very few villages had zero seroprevalence. There was a significant relationship between proportion seropositivity and being >15 km from the flood plains as compared to <5 km (Table 2).

**Table 2:** Animal level risk factors associated with RVF sero-positivity for cattle, goats and sheep in the Kilombero valley in multivariable analysis.

Variable	Levels	OR	95% CI
**Sex**	Female	1	
	Male	0.41	0.26–0.63
**Present during RVF outbreak**	No	1	
	Yes	4.67	3.33–6.55
**Distance to the edges of floods (km)**	<5 km	1	
	5–10 km	1.28	0.72–2.29
	10–15 km	0.89	0.48–1.65
	>15 km	1.92	1.04–3.56

OR = Odds ratio; CI = Confidence interval.

## Discussion

The findings from this serosurvey indicate post-epidemic and recent transmission of RVFV in livestock populations in the Kilombero valley. The demonstration of RVFV antibodies in animals as young as one year old, and the observed linear increase in the proportion of sero-converted animals from the age of 1 year to 5 years implies a constant exposure to infectious mosquito bites. The IgM antibodies detected in some animals illustrate presence of active foci of recent transmission preceding the serosurvey as the median duration of IgM antibodies to RVFV is two months [39]–[41]. These observations add to the increasing body of serological [3], [4], [42], [43] and virological [44], [45] evidence pertaining to RVFV transmission during the inter-epidemic periods in parts of Africa. Inter-epidemic transmission can be detected where active disease surveillance is in place in the livestock and/or human populations, as most of the inter-epidemic infections either are subclinical or mistaken for other diseases in the absence of public awareness of RVF presence [6], [46], [47].

The high prevalence observed in animals that were present during the outbreak is not surprising as during epidemics there is high exposure to RVFV with resulting high herd immunity [48], [49]. Such increased prevalence with age was also reported in sero-surveys in Madagascar, Nigeria and Senegal [47], [48], [50]. One of the few other studies reporting sex differences in RVF prevalence in livestock, a study in a slaughter house in Chad also found higher prevalence in female animals [51]. In contrast to our observation, a serosurvey in Madagascar reported higher prevalence in male animals [50]. Our observation might be as a result of female animals staying longer in a herd due to their role in reproduction and consequently most of sampled female animals were relatively older than their male counterparts. The different timing of our survey and the Madagascar survey, i.e. 4 years versus 3 months post-outbreak, might explain the different result on risk associated with sex in livestock populations.

Despite the sero-conversions observed in animals born after the 2006/07 outbreak, there have been no reports of epidemic or clinical disease in the area during the study period. This might be as a result of high herd immunity following the RVF outbreak as demonstrated by high prevalence in animals that were present during the previous epidemic in the study area. In such scenario high proportion of offspring born to immune dams would acquire maternal antibodies thus protected during vulnerable young age whereas old animals of local breeds are naturally less susceptible to clinical disease [52], [53]. Another explanation could be a circulation of non-virulent strain of RVFV [54] during inter-epidemic period in the area as it has been hypothesized in other reports of sero-conversion with no previous epidemic or clinical disease reports [46]. The sporadic cases of RVF could easily be confused with other livestock diseases which present similar clinical features of fever, including sporadic abortions and thus overlooked and under reported [53].

The contact between infected vectors and naïve mammalian hosts is the main determinant of arboviral disease transmission [55]. Mosquito vector population dynamics are driven by environment and ecology, which provide essential life resources. The trend observed in this study of increased seroprevalence away from the main flood plain and in high altitude is contrary to findings in other studies [6], [46]. This could be an indication of other factors playing a role including localized floods unrelated to the main flood area, but also proximity to dense vegetation (forested environments) that could harbor a variety of mosquito vectors [6], as the main forested areas are further away from the flood plain. Another explanation could be movement of previously exposed animals from one locality to another within the area through animal trade among livestock keepers, dowry or establishment of new households. In such circumstances, naïve animals can be transferred to an infected area or infected animals can be introducing the disease into a naïve location. In this way vector populations which are abundant and of diverse species within the valley [34] are exposed and maintenance mechanisms established where conditions are favorable. The observed separate hotspots further point to fine scale factors playing major role in transmission dynamics. On the other hand the only cold spot is located around Ifakara town, a semi urban environment in which livestock keeping is characterized by small herds, sedentary and mixed feeding practices as compared to relatively large herds with extensive system in the villages. The urban environment might be unfavorable to the main vector species but also due to limited daily livestock movements there is little interaction between neighbouring herds thus possibility of infection to limit itself to isolated herds in the event of disease outbreak.

Given the possible active transmission observed in this study within Kilombero valley and the moving out of livestock from the valley as a result of environmental degradation of wetland due to overstocking and overgrazing, there are chances for incubating or sick animals to introduce the disease into new areas. This might be enhanced by the quick means of transportation employed and possibilities for animals to harbour the RVFV for up to three weeks [52], [56]. In view of that, follow up of these moved herds and their new environment through serological and vector population monitoring will help to inform various stakeholders of the currently unidentified consequences, as livestock movement have been implicated to spread RVF in previously free areas [16], [57].

The interaction of livestock keepers with their animals is intense and includes milking, taking care of sick animals, grazing, using as draft animals, slaughtering and butchering and even children playing with animals. Future work should establish to what degree this inter-epidemic zoonotic circulation of RVFV leads to human infection as well. If considerable transmission to humans exists, health care providers within the Kilombero valley should consider RVF in their differential diagnosis in all fever cases presented in their facilities as RVF in humans may present with similar clinical signs to malaria, which is thought to be the main cause of fever in the valley [58]. We think this should be of priority in particular when dealing with patients from agro-pastoralist communities, especially when the malaria test is negative.

### Conclusion

The findings from this study indicate post-epidemic and recent transmission of RVFV in livestock populations in the Kilombero river valley. The linear increase in prevalence of RVFV antibodies in the post-epidemic annual cohorts implies a constant exposure and presence of active foci of recent transmission preceding the survey.

## Supporting Information

Table S1 Comparison of RVF prevalence across species, sex and presence during the 2006/07 RVF epidemic. (DOC)
