# Supplementary material for: Inter-epidemic Transmission of Rift Valley Fever in Livestock in the Kilombero River Valley, Tanzania: A Cross-Sectional Survey
Source: PLoS Negl Trop Dis. 2013 Aug 8;7(8):e2356. doi: 10.1371/journal.pntd.0002356 (PMC3738442; doi:10.1371/journal.pntd.0002356)
Supplement: Table S1 — (DOC) [file pntd.0002356.s001.doc]

**Table S1.** Comparison of RVF prevalence across species, sex and presence during the 2006/07 RVF epidemic

| **Presence during RVF outbreak** | **Sex** | **Species** | **Positive (n)** | **Prevalence** | **95% CI** |
| --- | --- | --- | --- | --- | --- |
| **Present** | Female | Cattle | 75 (304) | 24.67% | 19.93 – 29.91% |
|  |  | Goat | 30 (98) | 30.61% | 21.70 – 40.74% |
|  |  | Sheep | 15 (59) | 25.42% | 14.98 – 38.44% |
|  |  | *Sub-total* | 120 (461) | 26.03% | 22.08 – 30.29% |
|  | Male | Cattle | 6 (76) | 7.89% | 2.95 – 16.40% |
|  |  | Goat | 1 (19) | 5.26% | 0.13 – 26.03% |
|  |  | Sheep | 1 (8) | 12.50% | 0.32 – 52.65% |
|  |  | *Sub-total* | 8 (103) | 7.76% | 3.41 – 14.73% |
| **Born after** | Female | Cattle | 11 (306) | 3.59% | 1.81 – 6.34% |
|  |  | Goat | 21 (243) | 8.64% | 5.43 – 12.91% |
|  |  | Sheep | 10 (134) | 7.46% | 3.64 – 13.30% |
|  |  | *Sub-total* | 42 (683) | 6.14% | 4.46 – 8.22% |
|  | Male | Cattle | 15 (284) | 5.28% | 2.99 – 8.56% |
|  |  | Goat | 2 (95) | 2.11% | 0.26 – 7.40% |
|  |  | Sheep | 3 (54) | 5.56% | 1.16 – 15.39% |
|  |  | *Sub-total* | 20 (433) | 4.61% | 2.84 – 7.04% |
